# Comparison of clinical characteristics and hospital mortality in critically ill patients without COVID-19 before and during the COVID-19 pandemic: a multicenter, retrospective, propensity score-matched study

**DOI:** 10.1186/s13613-022-01028-2

**Published:** 2022-06-22

**Authors:** Sua Kim, Hangseok Choi, Jae Kyeom Sim, Won Jai Jung, Young Seok Lee, Je Hyeong Kim

**Affiliations:** 1 Department of Critical Care Medicine, College of Medicine, Korea University Ansan Hospital, Korea University, 123 Jeokkeum-ro, Danwon-gu, Ansan, 15520 Republic of Korea; 2 Medical Science Research Center, Korea University College of Medicine, Seoul, Korea; 3 Division of Pulmonology, Allergy and Critical Care Medicine, Department of Internal Medicine, Korea University Guro Hospital, Korea University, Seoul, Korea; 4 Division of Pulmonology, Allergy and Critical Care Medicine, Department of Internal Medicine, Korea University Anam Hospital, Korea University, Seoul, Korea

**Keywords:** Coronavirus disease 2019, Critically ill patients, Severity, Mortality, Propensity score matching

## Abstract

**Background:**

The high transmission and fatality rates of coronavirus disease 2019 (COVID-19) strain intensive care resources and affect the treatment and prognosis of critically ill patients without COVID-19. Therefore, this study evaluated the differences in characteristics, clinical course, and prognosis of critically ill medical patients without COVID-19 before and during the COVID-19 pandemic.

**Methods:**

This retrospective cohort study included patients from three university-affiliated tertiary hospitals. Demographic data and data on the severity, clinical course, and prognosis of medical patients without COVID-19 admitted to the intensive care unit (ICU) via the emergency room (ER) before (from January 1 to May 31, 2019) and during (from January 1 to May 31, 2021) the COVID-19 pandemic were obtained from electronic medical records. Propensity score matching was performed to compare hospital mortality between patients before and during the pandemic.

**Results:**

This study enrolled 1161 patients (619 before and 542 during the pandemic). During the COVID-19 pandemic, the Simplified Acute Physiology Score (SAPS) 3 and Sequential Organ Failure Assessment (SOFA) scores, assessed upon ER and ICU admission, were significantly higher than those before the pandemic (*p* < 0.05). The lengths of stay in the ER, ICU, and hospital were also longer (*p* < 0.05). Finally, the hospital mortality rates were higher during the pandemic than before (215 [39.7%] vs. 176 [28.4%], *p* < 0.001). However, in the propensity score-matched patients, hospital mortality did not differ between the groups (*p* = 0.138). The COVID-19 pandemic did not increase the risk of hospital mortality (odds ratio [OR] 1.405, 95% confidence interval [CI], 0.937–2.107, *p* = 0.100). SAPS 3, SOFA score, and do-not-resuscitate orders increased the risk of in-hospital mortality in the multivariate logistic regression model.

**Conclusions:**

In propensity score-matched patients with similarly severe conditions, hospital mortality before and during the COVID-19 pandemic did not differ significantly. However, hospital mortality was higher during the COVID-19 pandemic in unmatched patients in more severe conditions. These findings imply collateral damage to non-COVID-19 patients due to shortages in medical resources during the COVID-19 pandemic. Thus, strategic management of medical resources is required to avoid these consequences.

## Background

Coronavirus disease 2019 (COVID-19) is characterized by a hyperacute outbreak and high fatality due to respiratory failure [1, 2]. A substantial number of patients require admission to the intensive care unit (ICU) and mechanical ventilation [3–6], which causes a crisis in the health care system [7, 8]. Therefore, a shortage of medical resources during the COVID-19 pandemic has directly affected non-COVID-19 patients by delaying and disrupting appropriate treatment [9, 10]. In particular, critically ill non-COVID-19 patients have experienced fatal consequences, even under the relatively well-controlled severe acute respiratory syndrome coronavirus 2 (SARS-CoV-2) transmission conditions in Korea [11].

Since the first surge of COVID-19 in Daegu, the Korean healthcare system has been adapted to reduce the chance of exposure of non-COVID-19 patients to SARS-CoV-2 [12]. In hospitals, polymerase chain reaction (PCR) testing for SARS-CoV-2 is performed for patients with fever or respiratory symptoms at the emergency department (ED) and later, for all patients awaiting admission. COVID-19 patients are isolated from non-COVID-19 patients in the ED, general ward, and intensive care unit (ICU). To maintain this system, the Korean government has provided a substantial number of ICU beds for COVID-19 patients who need critical care in tertiary hospitals. However, these changes have caused a burden on the medical system for non-COVID-19 patients, and access to tertiary hospitals for non-COVID-19 patients has become increasingly difficult.

Higher severity and poor prognosis could be expected even in non-COVID-19 patients, given the burden on the medical system during this time. However, this effect has not been evaluated in countries with relatively effective control of the COVID-19 outbreak. Therefore, this study evaluated collateral damage to the critical care system during the COVID-19 pandemic in Korea. We compared the differences in patient characteristics, clinical course, and prognosis before and during the COVID-19 pandemic, and analyzed factors expected to influence the mortality of critically ill non-COVID-19 patients.

## Methods

### Study design

This was a multicenter retrospective cohort study. We screened and enrolled medical patients admitted to the ICU via the ED in three university-affiliated tertiary hospitals with 800–1200 beds in Seoul and the capital region, which accounted for > 60% of the daily COVID-19 incidence during the study period. These hospitals comprised 48–65 ICU beds, of which medical patients occupied 25–35 beds before the pandemic. However, the COVID-19 pandemic has caused the change of several systems in the emergency room (ER) and ICU of these hospitals, as well as patient characteristics, clinical course, and prognosis. We evaluated these changes due to the COVID-19 pandemic and analyzed the factors related to mortality in propensity score-matched patients.

### Study population

This study enrolled patients with critical medical conditions who were admitted to the ICU via the ER between January 1 and May 31, 2019, and between January 1 and May 31, 2021—after the government’s official order for increased ICU capacity for COVID-19 patients. Patients with surgical conditions or neurological issues, as well as those admitted to the ICU from the general ward, were excluded. Patients with SARS-CoV-2 infection were excluded from the study. Although ICUs are described as surgical, medical, cardiac, emergency, or neurocritical care units, they are run by an open system in which the boundary of each unit is dynamic. Therefore, eligible patients from any division of internal medicine were recruited during the study period.

### Data collection

Patient data were acquired from electronic medical records. We evaluated demographic parameters, medical history, comorbidities, severity scores calculated at ER and ICU admission (Simplified Acute Physiology Score [SAPS] 3 and Sequential Organ Failure Assessment [SOFA] score), and lengths of stay in the ER, ICU, and hospital. Organ support—including mechanical ventilation, continuous renal replacement therapy, and extracorporeal membrane oxygenation—and the presence of a do-not-resuscitate (DNR) order were investigated.

### Statistical analysis

Categorical variables are reported as numbers and frequencies (%). Continuous variables are presented as mean ± standard deviation (SD) and median (interquartile range [IQR]). Differences in frequencies were compared using the chi-squared test. Differences in continuous variables were compared using the *t* test, Mann–Whitney test, and analysis of variance. Hospital mortality was compared between propensity score-matched patients. Considering the possibility of a non-linear relationship between age and mortality, we divided the patient group by 70 years using Youden’s index in the logistic regression analysis. Included in the multivariate logistic regression model were the groups from before and during the COVID-19 pandemic period and factors related to hospital mortality from the univariate logistic regression analysis, including age > 70 years, Charlson comorbidity score, SAPS 3, SOFA score at ICU admission, and cause of ICU admission. In propensity score analysis, 1084 patients were included; patients with missing values were excluded in this analysis. Propensity score was calculated using logistic regression considering patient characteristics of age, Charlson comorbidity index, cause of admission, length of ER stay, presence of a DNR order, and SAPS3. Then, in each block for men and women after exact matching for sex, 1:1 propensity score matching was performed with the nearest neighbor matching method without replacement. The caliper width for the matching was 0.25 of the standard deviation of the logit of the propensity score. Statistical significance was set at *p* < 0.05. Statistical analyses were performed using SPSS Statistics (version 20.0; IBM Corp., Armonk, NY) and R 4.1.2 (The R Foundation for Statistical Computing, Vienna, Austria) for windows.

## Results

### Baseline patient characteristics

**Fig. 1:**
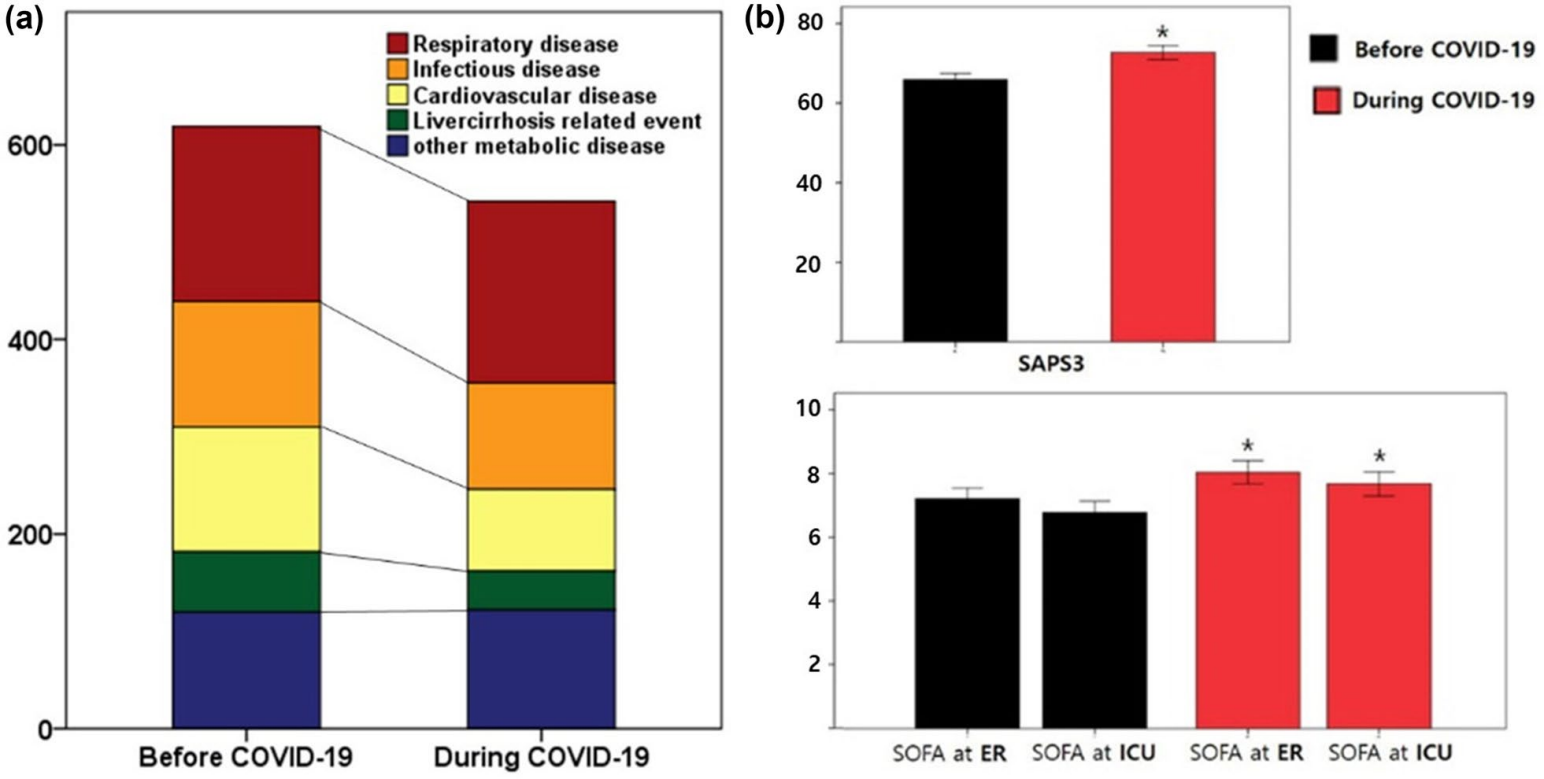
Patient numbers and their causes of admission (**a**) and severity at admission (**b**) before and during the COVID-19 pandemic. *p < 0.05, compared before and during the COVID-19 pandemic. COVID-19, coronavirus disease 2019

This study enrolled a total of 1161 patients, including 619 patients who were admitted to the ICU between January 1 and May 31, 2019, and 542 who were admitted to the ICU between January 1 and May 31, 2021 (Fig. 1a). The mean patient age was 68.1 ± 15.7 years, and 739 (63.7%) patients were men. These patients had at least one comorbidity, with a mean Charlson comorbidity score of 4.2 ± 1.9. The severity of the patients’ conditions, as evaluated in the ER using the SAPS 3 and SOFA scores, was 69.1 ± 19.7 and 7.6 ± 4.2, respectively. The most common cause of admission was respiratory disease except for COVID-19 (367, 31.6%), followed by sepsis (238, 20.5%) and cardiovascular disease (212, 18.3%) (Table 1).

**Table 1:** Baseline characteristics of the medical patient admitted to ICU before and during COVID-19 pandemic

	Total patients before propensity score matching				Propensity score matched patients			
	All patients (*n* = 1161)	Before COVID-19 pandemic (*n* = 619)	During COVID-19 pandemic (*n* = 542)	*p* value	All patients (*n* = 794)	Before COVID-19 pandemic (*n* = 397)	During COVID-19 pandemic (*n* = 397)	*p* value
Age (years), mean ± SD	68.1 ± 15.7	67.8 ± 15.1	68.6 ± 16.3	0.441	68.3 ± 15.8	68.7 ± 15.2	67.9 ± 16.4	0.483
Sex (male, %)	739 (69.7)	396 (64.0)	343 (63.3)	0.807	298	149	149	1.00
Charlson comorbidity score	4.2 ± 1.9	4.18 ± 1.90	4.16 ± 1.97	0.859	4.10 ± 1.96	4.10 ± 1.89	4.10 ± 1.93	0.970
Comorbidities								
Diabetes mellitus (%)	471 (40.5)	244 (39.4)	227 (41.9)	0.452	318	156	162	0.547
Hypertension (%)	627 (54.0)	328 (53.0)	299 (55.2)	0.483	435	213	222	0.521
Cardiovascular disease (%)	307 (26.4)	162 (26.2)	145 (26.8)	0.842	201	90	111	0.102
COPD/Asthma (%)	107 (9.2)	57 (9.2)	50 (9.2)	1.00	61	30	31	1.00
Liver cirrhosis (%)	67 (5.8)	41 (6.6)	26 (4.8)	0.208	43	20	23	0.754
Chronic kidney disease (IV, V) (%)	118 (10.2)	63 (10.1)	55 (10.1)	1.00	85	40	45	0.646
Malignancy (%)	168 (14.5)	106 (17.1)	62 (11.4)	0.007	102	59	43	0.111
Cerebrovascular disease (%)	197 (17.0)	109 (17.6)	88 (16.2)	0.583	137	75	62	0.260
Severity indices								
SAPS 3 (mean ± SD, Median [IQR])	69.1 ± 19.7 68 (54–83)	65.9 ± 18.6 64 (52–77)	72.7 ± 20.3 72 (57–87)	< 0.001	70.8 ± 8.5 70 (56–84)	71.0 ± 18.7 70 (56–84)	70.5 ± 19.7 70 (56–84)	0.712
SOFA score on ER admission (mean ± SD, Median [IQR)]	7.6 ± 4.2 7 (5–10)	7.2 ± 4.2 7 (4–10)	8.1 ± 4.2 8 (5–11)	< 0.001	7.8 ± 4.2 7 (5–11)	7.9 ± 4.2 7 (5–11)	7.8 ± 4.2 7 (5–11)	0.899
SOFA score on ICU admission (mean ± SD, Median [IQR)])	7.2 ± 4.4 7 (4–10)	6.8 ± 4.4 6 (4–9)	7.7 ± 4.4 7 (4–10)	0.001	7.6 ± 4.5 7 (4–10)	7.6 ± 5.6 7 (4–11)	7.5 ± 4.5 7 (4–10)	0.975
Cause of admission								
Respiratory disease (%)	367 (31.6)	180 (29.1)	187 (34.5)	0.050	261 (32.9)	129 (32.5)	132 (33.2)	0.880
Sepsis (except pneumonia) (%)	238 (20.5)	129 (20.8)	109 (20.1)	0.071	162 (20.4)	79 (19.9)	83 (20.9)	0.792
Cardiovascular disease (%)	212 (18.3)	128 (20.7)	84 (15.5)	0.027	137 (17.3)	69 (17.4)	68 (17.1)	1.00
Liver cirrhosis related event (%)	102 (8.8)	62 (10.0)	40 (7.4)	0.120	57 (7.2)	28 (7.1)	29 (7.3)	1.00
Other (DKA, pancreatitis, etc.) (%)	242 (20.8)	120 (19.4)	122 (22.5)	0.193	177 (22.2)	92 (23.2)	85 (21.4)	0.609

COVID, corona virus disease; ICU, intensive care unit; COPD, chronic obstructive pulmonary disease; SAPS, simplified acute physiology score; SOFA, sequential organ failure assessment; DKA, diabetic ketoacidosis

### Comparisons of patient characteristics and clinical course before and during the COVID-19 pandemic

Age, sex, and comorbidities did not differ significantly between the groups. However, the proportion of patients in each cause of admission changed (*p* = 0.031). The proportion of patients with respiratory disease increased during the COVID-19 pandemic (180 [29.1%] before vs. 187 [34.5%] during the pandemic, *p* = 0.050). The proportion of patients with other diseases decreased or remained unchanged. The severity indices of patients were significantly higher during than before the COVID-19 pandemic. The SAPS 3 was 65.9 ± 18.6 and 72.7 ± 20.3 (*p* < 0.001), the SOFA score on ER admission was 7.2 ± 4.2 and 8.1 ± 4.2 (*p* < 0.001), and the SOFA score on ICU admission was 6.8 ± 4.4 and 7.7 ± 4.4 (*p* = 0.001) before and during the pandemic, respectively (Table 1, Fig. 1b).

Compared to before the pandemic, the lengths of stay during the COVID-19 pandemic were longer in the ER (6.10 [4.11–9.58] h vs. 10.21 [7.21–19.43] h, *p* < 0.001), ICU (4 [2–7] days vs. 5 [2–10] days, *p *< 0.001), and hospital (10 [4–19] days and 12 [5–22] days, *p* = 0.044). Furthermore, more patients were intubated and mechanically ventilated (261 [42.2%] vs. 303 [55.9%], *p* < 0.001), and more patients signed a DNR order (111 [17.9%] vs. 141 [26.0%], *p* = 0.002) (Table 2).

**Table 2:** Clinical course of the patients before and during COVID-19 pandemic

	Total patients before propensity score matching			Propensity score matched patients		
	Before COVID-19 pandemic (*n* = 619)	During COVID-19 pandemic (*n* = 542)	*p* value	Before COVID-19 pandemic (*n* = 397)	During COVID-19 pandemic (*n* = 397)	*p* value
Organ support						
Mechanical ventilator (%)	261 (45.2)	303 (55.9)	< 0.001	210 (52.9)	209 (52.6)	1.00
Continuous renal replacement therapy (%)	118 (19.1)	125 (22.5)	0.096	90 (22.7)	89 (22.4)	1.00
Extracorporeal membrane oxygenation (%)	14 (2.3)	18 (3.3)	0.286	11 (2.8)	15 (3.8)	0.551
Duration of hospitalization						
ER length of stay (hours) (Mean ± SD, Median [IQR)])	8.70 ± 8.26 6.10 (4.11–9.58)	14.84 ± 13.30 10.21 (7.21–19.43)	< 0.001	10.14 ± 9.44 6.90 (4.52–11.24)	12.13 ± 9.96 9.08 (6.49–14.22)	0.004
ICU length of stay (days) (Mean ± SD, Median [IQR)])	5.6 ± 7.7 4 (2–7)	8.0 ± 9.3 5 (2–10)	< 0.001	6.6 ± 7.8 4 (2–8)	7.7 ± 9.0 5 (2–9)	0.060
Hospital length of stay (days) (Mean ± SD, Median [IQR])	15.8 ± 20.0 10 (4–19)	16.5 ± 16.7 12 (5–22)	0.044	16.7 ± 20.0 10 (4–22)	15.9 ± 16.0 11 (5–21)	0.589
Limitation of the treatment						
Do-Not-Resuscitation (DNR) order (%)	111 (17.9)	141 (26.0)	0.002	87 (21.9)	102 (25.7)	0.243
Limitation of the treatment (%)*	81 (13.1)	111 (20.5)	0.001	57 (14.4)	76 (19.1)	0.087
DNR in hopeless patient (%)	38 (6.1)	38 (7.0)	0.555	32 (8.1)	26 (6.5)	0.496
ICU mortality (%)	154 (24.9)	199 (36.7)	< 0.001	121 (30.5)	141 (35.5)	0.151
Hospital mortality (%)	176 (28.4)	215 (39.7)	< 0.001	131 (33.0)	152 (38.3)	0.138

COVID, corona virus disease; ER, emergency room; SD, standard deviation; IQR, interquartile range; ICU, intensive care unit

^*^ The number of patient who refused intubation, renal replacement therapy, transfusion, vasoactive agent or chemotherapy

### Length of stay in the ER and changes in patient condition during emergency department care

**Fig. 2:**
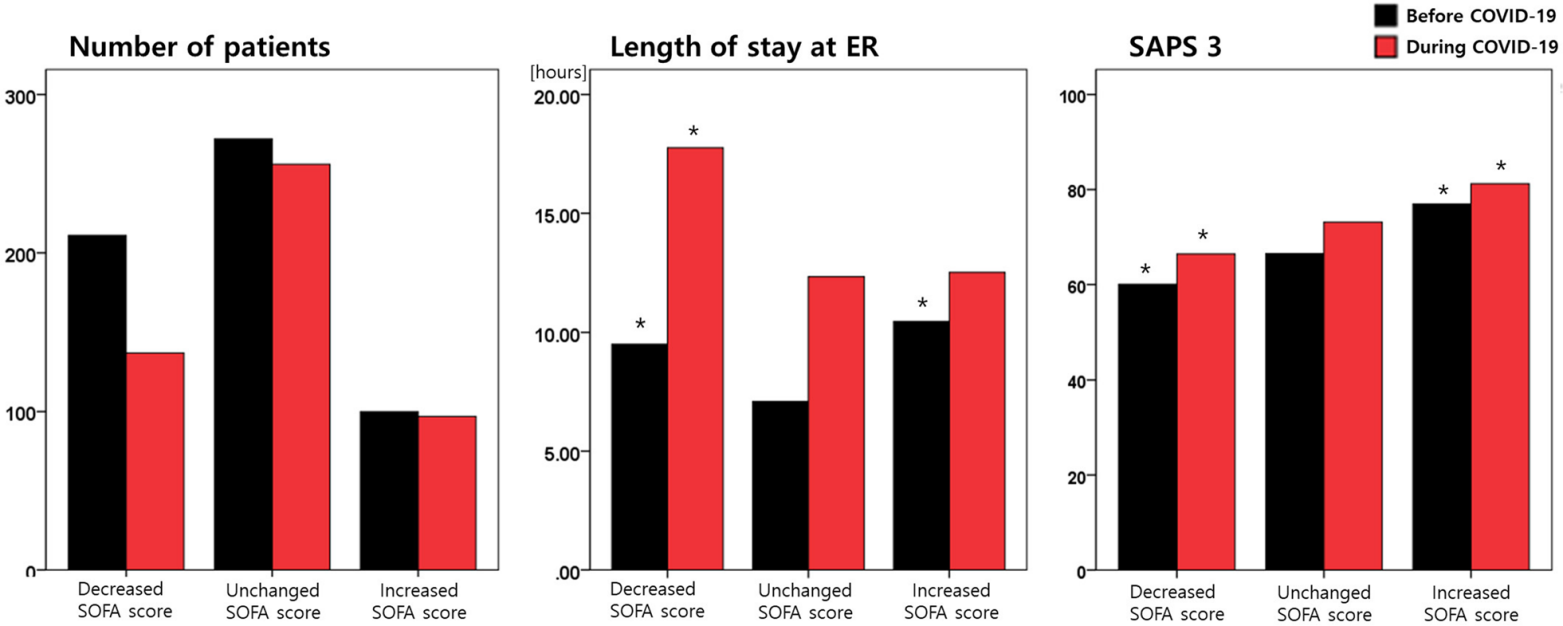
Characteristics of patients by changes in SOFA scores during emergency department care. (**a**) Patient numbers, (**b**) length of stay in the ER, and (**c**) SAPS 3 in each group. *p < 0.05, compared to the values in the patient group with unchanged SOFA scores. SOFA, Sequential Organ Failure Assessment; ER, emergency room; SAPS 3, Simplified Acute Physiology Score, version 3

We evaluated the changes in patient condition during ED care based on the changes in SOFA scores upon ER admission and ER discharge (or upon ICU admission) in each patient. Patients were then divided into three groups: those whose condition improved (decrease in SOFA score), those whose condition remained unchanged (unchanged SOFA score), and those whose condition deteriorated (increase in SOFA score). Prolonged ER stay was not related to the deterioration of patient condition during ED care; ER stay was longer in patients whose condition improved than in patients whose condition remained unchanged (Fig. 2b). Changes in conditions during ED care were significantly related to SAPS 3. Patients whose conditions deteriorated during ED care had significantly higher SAPS 3 than patients with unchanged or improved conditions, both before and during the COVID-19 pandemic (SAPS 3 of patients with an improved, unchanged, and deteriorated condition, respectively: 60.1 ± 16.8 vs. 66.5 ± 17.6 vs. 76.9 ± 20.0, *p* < 0.001, before the pandemic; 66.4 ± 18.9 vs. 73.1 ± 19.6 vs. 81.2 ± 22.1, *p* < 0.001, during the COVID-19 pandemic.) (Fig. 2c).

### COVID-19 pandemic and hospital mortality in propensity score-matched critically ill non-COVID-19 patients

Propensity score matching identified 397 patients in each group before and during the pandemic. Differences in baseline characteristics and clinical course, including severity indices, were minimized or disappeared after matching (Tables 1 and 2). In propensity score-matched patients, ICU and hospital mortality rates did not differ between the groups (121 [30.5%] and 141 [33.5%], *p* = 0.151; 131 [33.0] and 152 [38.3], *p* = 0.138). In the univariate logistic regression analysis, hospital mortality was related to age > 70 years, Charlson comorbidity score, cause of admission, SAPS 3, SOFA score upon ER and ICU admission, and presence of a DNR order. Both the length of stay in the ER (odds ratio [OR] 0.989, 95% confidence interval [CI] 0.974–1.005, *p* = 0.167) and the COVID-19 pandemic (OR 1.260, 95% CI 0.942–1.685, *p* = 0.120) were not associated with hospital mortality. In the multivariate logistic regression analysis, risk of hospital mortality was increased by SAPS 3 (OR 1.065, 95% CI 1.048–1.081, *p* < 0.001), SOFA score at ER admission (OR 1.148, 95% CI 1.078–1.224, *p* < 0.001), and presence of a DNR order (OR 8.160, 95%CI 5.072–13.130, *p* < 0.001) (Table 3). The COVID-19 pandemic had no impact on hospital mortality in propensity score-matched critically ill patients in the multivariate logistic regression analysis (OR 1.405, 95% CI, 0.937–2.107, *p* = 0.100).

**Table 3:** Univariate and Multivariate Cox proportional hazard regression analysis for in-hospital mortality in propensity score matched patients

	Univariate logistic regression analysis			Multivariate logistic regression analysis		
	Odds ratio	95% confidence interval	*p* value	Odds ratio	95% confidence interval	*p* value
Age > 70	1.779	1.325–2.389	< 0.001	0.758	0.457–1.259	0.284
Sex	0.885	0.654–1.196	0.425			
Charlson comorbidity index	1.266	1.166–1.374	< 0.001	1.099	0.962–1.256	0.165
SAPS 3	1.089	1.075–1.103	< 0.001	1.065	1.048–1.081	< 0.001
SOFA on ER admission	1.322	1.261–1.386	< 0.001	1.148	1.078–1.224	< 0.001
SOFA on ICU admission	1.376	1.311–1.444	< 0.001			
Cause of admission			< 0.001			0.286
Respiratory disease	3.032	1.930–4.762	0.001	1.091	0.591–2.011	0.781
Cardiovascular disease	1.929	1.142–3.259	0.014	1.827	0.901–3.708	0.095
Liver cirrhosis	3.660	1.922–6.969	< 0.001	0.918	0.355–2.378	0.861
Infectious disease	3.762	2.308–6.130	< 0.001	1.520	0.798–2.896	0.202
Other metabolic	Reference			Reference		
ER length of stay	0.989	0.974–1.005	0.167			
Do-not-resuscitation order	10.728	7.303–15.760	< 0.001	8.160	5.072–13.130	< 0.001
COVID-19 pandemic	1.260	0.942–1.685	0.120	1.405	0.937–2.107	0.100

SAPS 3, simplified acute physiology score 3; SOFA, sequential organ failure assessment; ER, emergency room; COVID-19, corona virus disease 2019

## Discussion

We compared patient characteristics, clinical course, and mortality in non-COVID-19 critically ill patients before and during the COVID-19 pandemic. The baseline characteristics of these patients did not differ except for the cause of admission. During the COVID-19 pandemic, the severity of conditions was higher, the lengths of stay in the ER, ICU, and hospital were longer, and the ICU and hospital mortality rates were higher. However, hospital mortality did not differ between the propensity score-matched groups of patients before and during the pandemic. Only SAPS 3, SOFA score, and presence of a DNR order increased the risk of hospital mortality in the multivariate logistic regression analysis, whereas COVID-19 did not.

Korea is a country in which SARS-CoV-2 transmission is relatively well-controlled compared to other countries [12–14]. The daily incidence of COVID-19 in our country was 527 ± 85, and < 300 COVID-19 patients required ICU care in tertiary hospitals during the study period. However, we have experienced a shortage of intensive care resources in the ER and ICU [15, 16], which has affected not only COVID-19 patients but also non-COVID-19 patients [17]. Since the first patients were diagnosed with COVID-19 and the first surge of the disease occurred in Korea, the emergency medical system has changed. Patients with respiratory symptoms and/or fever were separated from other patients in the ER. This limited the capacity of the ER for patients with respiratory and/or infectious diseases who required medical services. A PCR test for SARS-CoV-2 was routine for all patients awaiting admission, which significantly increased the length of their stay in the ER. Moreover, temporary closure of certain zones of the ER that had been exposed to SARS-CoV-2 was common. Therefore, the turnover rate of beds in the ER decreased. The conditions of the ICU and intensive care system also changed. With the increase in the number of critically ill COVID-19 patients, many ICUs were allocated to patients with this contagious disease in isolation, according to government order; 1% of the total bed number was assigned to the COVID-19 ICUs in December 2020, which became 4% when the daily incidence exceeded 5000 cases [18]. Because COVID-19 patients were admitted to units completely isolated from non-COVID patients, these beds were not available to non-COVID-19 patients even when they were unoccupied. Although additional beds and facilities for critically ill COVID-19 patients were gradually provided, the number of ICU beds for non-COVID-19 patients decreased by 10 to 15 in each hospital included in this study during the study period. Furthermore, intensivists and critical care nurses were insufficient in number and difficult to recruit; therefore, the workload of these experienced practitioners increased [8, 19].

Outbreaks of infectious diseases can change the patterns of ER visits. More patients ignore significant medical signs and symptoms [9, 20]. However, the proportion of patients with febrile symptoms increased during the outbreak, while neither the proportion of critically ill patients—nor their mortality rates—changed [21, 22]. Our results showed a pattern similar to that reported previously. The proportion of patients with cardiovascular disease decreased, and the proportion of patients with respiratory disease increased during the pandemic, along with an increase in clinical severity.

Prolonged stays in the ER are known to influence patient mortality, owing to the shortage of critical care resources, including staff and equipment [23]. However, in our study, increased length of stay in the ER influenced the prognosis of the patients differently. Although prolonged stay in the ER did not directly impact the prognosis of patients, it increased the overall ER census and the threshold of ER admission. The decreased number of ICU beds and increased length of ICU stay during the pandemic also delayed ICU admission from the ER and caused prolonged stay in the ER, further increasing the ER census. Therefore, patients visited the ER in worse condition during the pandemic than those before, and the greater severity of critically ill patients at ER visit may have influenced their prognosis during this pandemic.

Owing to the large proportion of COVID-19 patients presenting with acute respiratory failure requiring critical care, the shortage of medical resources for critical care is expected. In this COVID-19 crisis, resource limitations impacted outcomes [24, 25]. Therefore, strategies for efficient allocation of available resources are essential. However, because the end of the crisis were not visible, a strategy is urgently needed for the efficient use of medical resources, including space, staff, and equipment [26].

This study had several limitations. First, we evaluated only patients admitted to the ICU via the ER. Our data did not include patients who were not admitted to the ICU from the ER, even those with high disease severity. Therefore, the results of this study cannot represent all critically ill patients who visit the ER. Second, the policy for managing patients in the ER differed among hospitals, which may have influenced the length of stay in the ER. Therefore, we calculated propensity score considering the length of stay in the ER and evaluated the association between the length of stay in the ER and patient mortality in propensity score-matched patients. Third, we screened patients during the same period in 2019 and 2021, considering the seasonal variance in the characteristics of patients admitted to the ICU. However, this study was not designed to investigate improvements in patient care and outcomes over 2 years. Fourth, we did not measure the difference in the quality of ICU care before and during the pandemic, which may have significantly influenced patient outcomes. Finally, due to the retrospective cohort design of the study, we can only comment on association of factors and not on causation of mortality of critically ill patients during the pandemic.

## Conclusions

During the COVID-19 pandemic, the severity of patient conditions upon admission, as well as hospital mortality, were significantly higher than before the pandemic. However, in the propensity score-matched groups with similar severity indices, hospital mortality did not differ between the groups. The risk of hospital mortality in critically ill non-COVID-19 patients was not related to the COVID-19 pandemic, but to the severity indices of the patients and the DNR status. Although further study is needed, delayed access to medical services may be related to higher severity and mortality in non-COVID-19 critically ill patients. Establishing a strategy to manage medical resources is needed to halt the interaction between delayed access to medical services and poor outcomes in critically ill patients.

## Data Availability

The data set supporting the results of this study is available from the corresponding author upon reasonable request.

## Abbreviations

COVID-19: Coronavirus disease-2019
SARS-CoV-2: Severe acute respiratory syndrome coronavirus 2
ICU: Intensive care unit
ER: Emergency room
ED: Emergency department
SAPS 3: Simplified Acute Physiology Score, version 3
SOFA: Sequential Organ Failure Assessment
DNR: Do-not-resuscitate
SD: Standard deviation
IQR: Interquartile range
CI: Confidence interval
